# Differential Brain Perfusion Changes Following Two Mind–Body Interventions for Fibromyalgia Patients: an Arterial Spin Labelling fMRI Study

**DOI:** 10.1007/s12671-021-01806-2

**Published:** 2022-01-05

**Authors:** Sonia Medina, Owen G. O’Daly, Matthew A. Howard, Albert Feliu-Soler, Juan V. Luciano

**Affiliations:** 1 Department of Neuroimaging, King’s College London, London, UK; 2 Department of Clinical & Health Psychology, Autonomous University of Barcelona, Bellaterra, Spain; 3 Research & Innovation Unit, Parc Sanitari Sant Joan de Déu, Sant Boi de Llobregat, Spain

**Keywords:** Fibromyalgia, Mindfulness, Meditation, pCASL fMRI

## Abstract

**Objectives:**

Further mechanistic insight on mind–body techniques for fibromyalgia (FMS) is needed. Arterial spin labelling (ASL) imaging can capture changes in regional cerebral blood flow (rCBF) that relate to spontaneous pain.

**Methods:**

We recruited FMS patients undergoing either mindfulness-based stress reduction training (MBSR, *n* = 14) or a psychoeducational programme (FibroQoL, *n* = 18), and a control FMS group with no add-on treatment (*n* = 14). We acquired whole-brain rCBF maps and self-report measures at baseline and following treatment and explored interaction effects in brain perfusion between the treatment group and session with a focus on the amygdala, the insula and the anterior cingulate cortex (ACC).

**Results:**

We identified a significant interaction effect in the amygdala, which corresponded with rCBF decreases following FibroQoL specifically. At baseline, rCBF in the amygdala for the FibroQoL group correlated with pain catastrophizing and anxiety scores, but not after treatment, suggesting a decoupling between activity in the amygdala and negative emotional symptoms of FMS as a consequence of treatment. Baseline rCBF correlated positively with pain symptoms in the ACC and the anterior insula across all patients; moreover, the correlation between rCBF changes post intervention in the insula and pain improvement was negative for both treatments and significantly different from the control group. We suggest that there is disruption of the typical relationship between clinical pain and activity as a product of these two nonpharmacological therapies.

**Conclusions:**

We have demonstrated that different mind-to-body treatments correspond to differential changes in clinical symptoms and brain activity patterns, which encourages future research investigating predictors of treatment response.

**Trial Registration:**

NCT02561416.

**Supplementary Information:**

The online version contains supplementary material available at 10.1007/s12671-021-01806-2.

Fibromyalgia syndrome (FMS) is a chronic condition characterized by widespread musculoskeletal pain, fatigue, stiffness, sleep disturbances, cognitive problems and distress (Häuser et al., 2015). Because of a lack of consistency of diagnostic criteria (Goldenberg, 2009), the high heterogeneity of early symptoms and unknown primary cause of this condition, FMS diagnosis is very often by exclusion, long after onset or following prior misdiagnoses (Wolfe et al., 2019). Given the broad spectrum of symptoms present in FMS, it is crucial to adopt a multidisciplinary approach to manage FMS at all stages of the disorder (Nüesch et al., 2013). Despite this, standard treatments available have traditionally only targeted alleviation of pain symptoms with only modest success, namely tricyclic antidepressants, opioids, sedatives, tramadol and nonsteroidal anti-inflammatory drugs (Sumpton & Moulin, 2008). More recently, nonpharmacological interventions have started to be incorporated as part of standard clinical care, including physical therapy (Stucki, 2000) or cognitive behavioural therapy (Bennett & Nelson, 2006), mindfulness (Aman et al., 2018; Pérez-Aranda, D’Amico, et al., 2019), virtual reality therapy (Garcia-Palacios et al., 2015) or clinical hypnosis (Picard et al., 2013). However, little-to-no knowledge exists regarding their mechanisms of action within the central nervous system. Accordingly, to date, biomarkers that facilitate treatment response prediction have yet to be developed (Häuser et al., 2015) in order to promote individualized treatment plans for patients.

Neuroimaging techniques are useful tools to study mechanisms of action of nonpharmacological interventions for clinical pain (Jensen et al., 2012a, b, c), specifically studies employing functional magnetic resonance imaging (fMRI) using blood oxygen level-dependent (BOLD) contrasts. They have provided evidence of altered functional connectivity (FC) of the default mode network (DMN) (Fallon et al., 2016) and cognitive control network in FMS patients compared to healthy controls (Kong et al., 2018), as well as changes in the aforementioned networks following transcranial direct current stimulation to the motor cortex (Cummiford et al., 2016), cognitive behavioural therapy (Jensen et al., 2012a, b, c), hypnosis (Derbyshire et al., 2009) or mindfulness (Young et al., 2018). The amygdala seems to play a key role in encoding major targets of psychological therapies for chronic pain, such as depression (Giesecke et al., 2005) or anxiety and fear of pain (Hsiao et al., 2020). Studies using sustained-pain BOLD fMRI paradigms point towards FC increases of the somatosensory cortex with the insula during pain in FMS patients (Kim et al., 2015) which can be altered with cognitive therapy (Lazaridou et al., 2017); they also suggest that there is less FC within the pain inhibitory network during pressure pain, using the rostral anterior cingulate cortex (ACC) as seed (Jensen et al., 2012a, b, c). The subgenual ACC (sgACC) is an important hub for descending pain control and analgesia (Schrepf et al., 2016; Sprenger et al., 2011), and has previously shown deficient activation during pain tasks in FMS patients (Jensen et al., 2009). Moreover, the insula and the ACC are directly linked via the salience network, which is essential for cognitive control and holds a complementary function with the DMN (Jilka et al., 2014). Nevertheless, as Jensen et al., (2012a, b, c) suggested, results from traditional fMRI studies relate to experimental paradigms optimized in the lab, where BOLD fMRI techniques rely on rapid, higher frequency fluctuations in hemodynamic signals. BOLD fMRI is therefore not sufficiently sensitive to capture low-frequency, long-lasting fluctuations more closely related to spontaneous persistent pain and natural clinical improvement. Arterial spin labelling (ASL) is an fMRI technique that offers an alternative to BOLD via measuring cerebral blood flow (rCBF) across the brain, and is capable of measuring those slow subtle perfusion differences (Loggia et al., 2019). Although ASL presents a valuable opportunity to study how rCBF relates to FMS symptom improvement as well as mechanisms of action of treatments for FMS, the literature in this field is to date scarce (Moana-Filho et al., 2013; Müller et al., 2021; Zeidan et al., 2018). A comprehensive model of the mechanism of action of novel non-pharmacological techniques for FMS using perfusion fMRI is yet to be established.

The present study includes data from a subsample of FMS patients who took part in the EUDAIMON study (Pérez-Aranda, D’Amico, et al., 2019; Pérez-Aranda, Feliu-Soler, et al., 2019), a randomized, controlled trial (RCT) that assessed the efficacy and cost-utility of mind–body interventions to manage FMS symptoms: mindfulness-based stress reduction (MBSR), and a psychoeducational and relaxation training programme (FibroQoL) (Luciano, Aguado, et al., 2013; Luciano, Sabes-Figuera, et al., 2013). Both interventions are accepted as active treatments; they are equivalent in structure, produce similar reductions in FMS impact on patients’ daily lives in the long term (Pérez-Aranda, Feliu-Soler, et al., 2019) and demonstrate superior cost-utility than treatment as usual alone; however, whether the neurophysiological mechanisms of action of both treatments are comparable is yet to be assessed. We applied ASL fMRI to acquire rCBF maps from two groups of FMS patients prior to and following one of these interventions in addition to their treatment as usual (TAU). An additional control group of FMS patients who continued with TAU only was also recruited. We sought to determine (a) whether rCBF changed differentially between treatment groups, in order to better understand the mechanism of action of each treatment; (b) how rCBF changes relate to FMS symptom improvements and (c) the utility of rCBF as a viable biomarker of clinical pain in FMS. We hypothesized that rCBF in the amygdala would reduce following treatment, as a consequence of reduced stress and improved anxiety control (Jiang et al., 2016); furthermore, since mind–body interventions such as mindfulness training aim to improve cognitive control, emotional regulation and body awareness, we hypothesized that MBSR and FibroQoL would induce changes within the salience network via perfusion reductions at rest in insula and ACC following treatment.

## Methods

### Participants

A total of 90 FMS female patients recruited in this RCT participated in the neuroimaging sub-study. Due to image artefacts and missing imaging data in one of the study sessions and participant withdrawal, we included data from a total of only 46 patients in the final analysis. Participants were recruited from a database of 531 patients at the Rheumatology Service at Parc Sanitari Sant Joan de Déu, St. Boi de Llobregat (Spain). Patients had been previously diagnosed with FMS according to the *American College of Rheumatology 1990 Criteria* at the recruitment site. Inclusion criteria were the following: (i) female, right-handed patients between 18 and 65 years of age, (ii) able to understand Spanish language, (iii) willing to provide written consent to participate. Exclusion criteria included the following: (i) participation in other RCTs, (ii) presence of cognitive impairment according to the Mini-Mental State Examination (MMSE < 27), (iii) receiving psychological treatment during the previous or current year, (iv) previous experience in meditation or other mind–body therapies, (v) comorbid mental disorder or severe medical illness which may interfere with the study, (vi) inability to attend group sessions, (vii) pregnancy, (viii) involvement in ongoing litigation relating to FMS. Additional exclusion criteria for the neuroimaging substudy participation included (i) usual MRI contraindications (claustrophobia, metal implants, pacemakers, etc.), (ii) consuming more than 8 caffeine units per day (1 caffeine drink was permitted on the day of the study, (iii) smoking more than 5 cigarettes per day, (iv) acute pain not related to FMS on the day of the study (e.g. headache, lumbar pain). Participants were instructed to refrain from taking any rescue analgesic aside from TAU 72 h prior to each MRI session. Since patients were also evaluated for their immune-inflammatory status (results published in Andrés-Rodríguez et al. 2019), additional exclusion criteria were also taken into account for the present study: infection/cold symptoms on the day of blood extraction; needle phobia; BMI > 36 kg/m^2^ or weight > 110 kg; neoplastic illnesses, infection, cardiopulmonary, vascular, or other internal conditions (collected from the medical history); use of oral or local corticosteroids or anticytokine therapy.

### Procedure

**Fig. 1:**
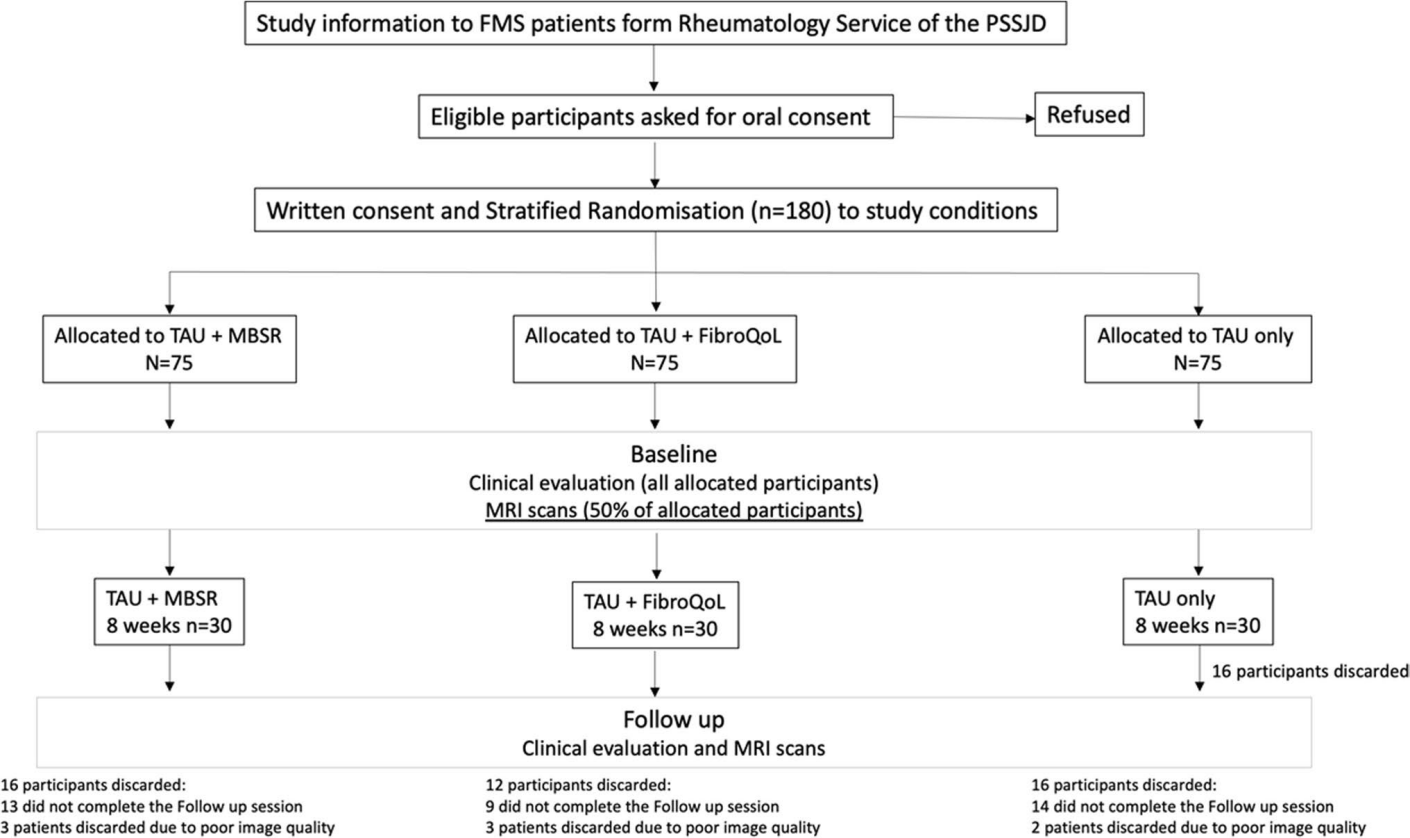
Flowchart of experimental design for the EUDAIMON neuroimaging substudy**.** Figure adapted from EUDAIMON study protocol

**Fig. 2:**
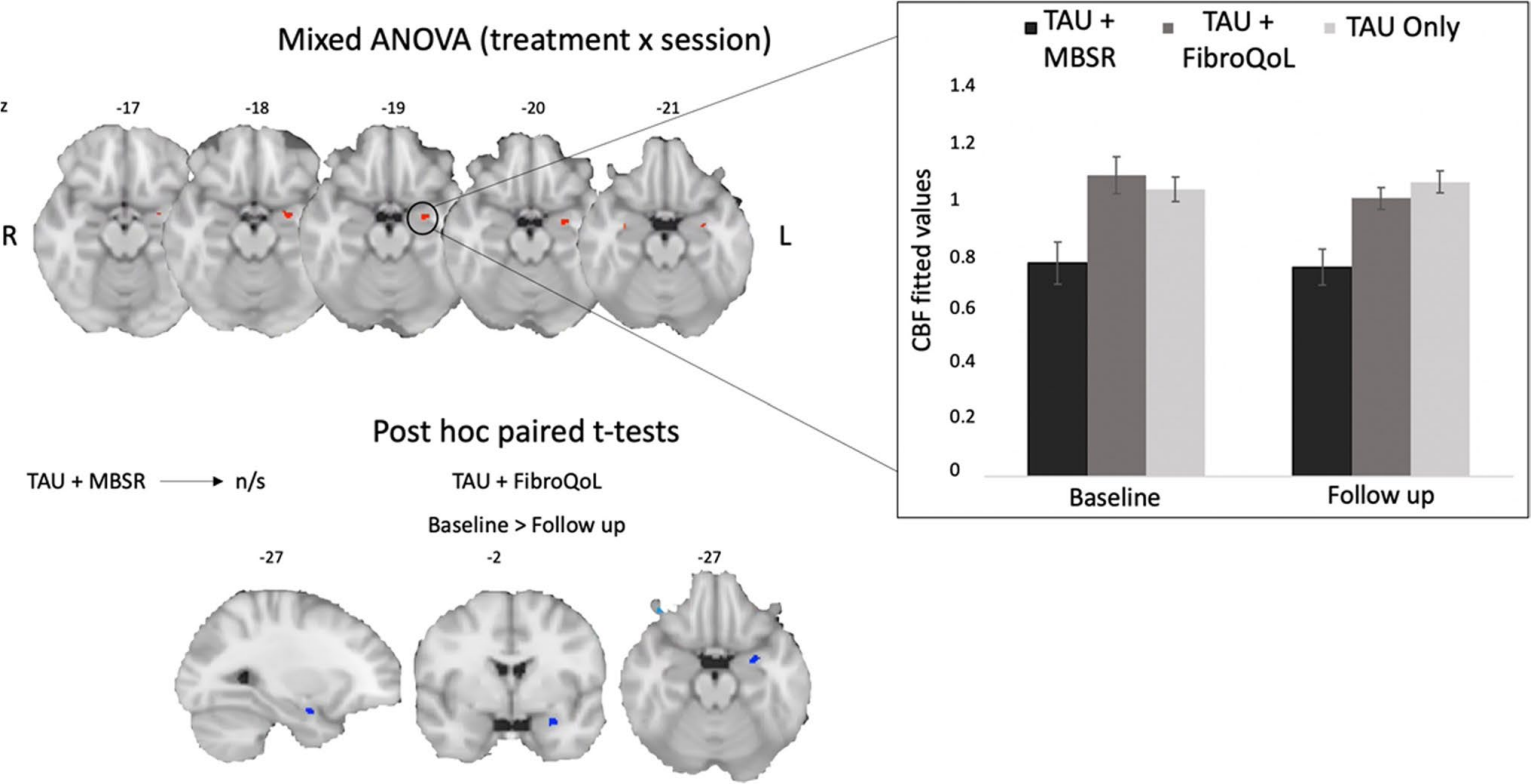
Results from mixed 3 by 2 ANOVA and post hoc pairwise comparisons. Initial cluster-forming threshold = 0.005. Results are significant at peak level after family-wise error correction at alpha = 0.05 post SVC in the amygdala

The present study was part of a 12-month, parallel-group, randomized, single-blind, controlled trial with three treatment arms, TAU + MBSR, TAU + FibroQoL, and TAU only. The Consolidated Standards of Reporting Trials 2010 (CONSORT; Schulz et al., 2010) were followed. Participants underwent an initial clinical evaluation to confirm diagnosis and eligibility. Eligible participants were randomly allocated to one of the three treatment groups (i.e. TAU, TAU + MBSR, and TAU + FibroQoL), and from there, they were assigned to either the main RCT or the RCT + neuroimaging substudy. Participants attended a baseline clinical evaluation blind to treatment allocation. In the next 5 days, all patients from the neuroimaging substudy were contacted to complete a baseline MRI scanning session. Following their first scanning session, participants then underwent their allocated study intervention for the following 8 weeks; once the treatment phase was finalized, participants attended a follow-up visit, where an identical clinical evaluation and MRI scanning were carried out (for a flowchart of the study design, see study protocol (Feliu-Soler et al., 2016) and Fig. 1 and 2).

The following interventions were administered:

#### Intervention Group 1 (TAU + MBSR)

Participants underwent the MBSR protocol developed at the University of Massachusetts Medical School, USA, consisting of 8 weekly 2-h-long group sessions (*n* = 15) of mindfulness training. MBSR was developed to help people with chronic pain and other stress-related conditions (Kabat-Zinn, 1982). To avoid exhaustion or withdrawal from the study, session duration was slightly shortened (2 instead of 2.5 h). Additionally, we offered patients an optional half-day of silent MBSR retreat between weeks 6 and 7. Both the group and the homework sessions included elements of MBSR practice such as body scan, sitting meditation and mindful movements/stretches, with the purpose of helping patients to relate to their physical and psychological symptoms in more accepting and non-judgmental ways. Patients were provided with a MBSR book (Martin-Ausero, 2008).

#### Intervention Group 2 (TAU + FibroQoL)

FibroQoL was structurally equivalent to MBSR (8 weekly 2-h-long sessions) and consisted of a group-based (*n* = 15) psycho-educational programme specific to FMS patients, led by specialized clinicians, divided into two parts: 4 sessions of psychoeducation in which patients receive information about the pathophysiology, diagnosis and management of FM symptoms and another 4 sessions of training in self-hypnosis to generate a state of deep relaxation with the objective of achieving control over the body and pain (a focus that is inconsistent with mindfulness) and to imagine one’s life in the future without pain.

Patients allocated in the active treatment arms were instructed to continue their usual pattern of medication and recorded their usual care over the course of the RCT. Audiotapes were also provided to both groups to facilitate practice at home.

#### Treatment as Usual Control Group (TAU)

The TAU provided was mainly pharmacological as per usual Spanish clinical practice, accompanied by counselling on aerobic exercise according to individual patients’ physical conditions.

### Measures

The measures listed below were the ones included in the neuroimaging sub-study by the researchers, as they may contribute to explain inter-individual variability in the imaging data results. For a detailed summary of all the outcome measures, the reader is referred to the study protocol (Feliu-Soler et al., 2016).

#### Demographic and Clinical Measures

We collected the following information from participants: age, years from FMS diagnosis, pharmacological medication as part of their TAU and psychiatric comorbidities (e.g. depression).

#### Self-report Clinical Measures

In all clinical evaluations (i.e. baseline and follow-up sessions), participants completed a series of standardized questionnaires, in order to better characterize patients’ phenotypes and to assess changes in symptoms associated with FMS after treatment. These were (i) the Revised Fibromyalgia Impact Questionnaire (FIQR) (Bennett et al., 2009; Luciano, Aguado, et al., 2013; Luciano, Sabes-Figuera, et al., 2013), (ii) the Hospital Anxiety and Depression Scale (HADS) (Luciano et al., 2014; Zigmond & Snaith, 1983) and (iii) the Pain Catastrophizing Scale (PCS) (García-Campayo et al., 2010; Sullivan et al., 1995). Participants also rated their perceived pain intensity on the day of each scanning session using a paper-and-pencil horizontal, 100-mm visual analogue scale anchored with ‘no pain’ and ‘maximum pain’ (Langley & Sheppeard, 1985).

#### MRI Data Acquisition and Preprocessing

We performed MRI measurements on a 3.0-T Phillips Ingenia wide-bore MR scanner, equipped with an 8-channel, phased-array, receive-only head coil. All patients had an axial T2-weighted 3D turbo spin-echo (TSE) pulse sequence with slice thickness = 3 mm, repetition time = 3000 ms, echo time = 95 ms, flip angle = 90° and field of view = 240 × 180 × 125 mm. For brain perfusion measurements, we used a fast, single-shot, pCASL sequence (repetition time = 5000 ms, label distance = 90 mm, label duration = 1650 ms, post-labelling delay = 1600 ms, voxel size = 1.875 × 1.875 × 3 mm). We finally quantified a regional cerebral blood flow (rCBF) map per patient for each MRI session. For image preprocessing, we used the Statistical Parametric Mapping software (SPM) version 12. First, we segmented the structural T2 images and normalized the resulting grey matter (Zigmond and Snaith) masks to Montreal Neurological Institute (MNI) space using non-linear registration tools from the DARTEL toolbox in SPM. For this step, we created an intermediate study-specific template to avoid bias associated with group normalization. We then performed co-registration of the pCASL images to native-space T2 scans. The co-registered functional images were then warped into MNI space using the deformation parameters estimated from the normalization step of the T2 scans as well as the study-specific template. Finally, we multiplied each normalized functional image by a binary, skull-stripped T2 template in order to remove any extracerebral tissue from the images. We performed manual quality assurance at each step to identify artefacts (e.g. co-registration failures) as well as outliers. Halfway through MRI data acquisition an unexpected, unplanned update of the scanner software took place, causing the functional images acquired afterwards to be scaled differently. To mitigate this issue, we calculated the mean global rCBF for each image and divided each voxel by their respective mean global rCBF value, resulting in normalized, comparable rCBF maps.

### Data Analyses

#### Clinical Data

Once we checked that normality assumption was met, we compared baseline data across the three treatment groups via a one-way analysis of variance (ANOVA) with subsequent post hoc tests in order to ensure the groups were comparable. We also explored changes in each one of the self-report measures of interest following treatment via paired samples *t*-test. We performed these analyses in SPSS v. 26.

#### Neuroimaging Data

We adopted a mass univariate general linear model approach for all group-wise statistical analyses of the pCASL data. We set all initial cluster-forming height thresholds to *p* < 0.005 and applied family-wise error (FWE) correction at cluster extent *p* < 0.05. For each one of our regions of interest, which we examined via a small volume correction (SVC) for each contrast, we applied FWE correction at cluster peak level. The ROIs chosen for analyses were the amygdala bilaterally, the insula bilaterally, the pregenual ACC and the sgACC. We chose the insula and amygdala predefined masks from the WFU_PickAtlas Toolbox in SPM. The pregenual and sgACC ROIs were extracted from the Brainnetome Atlas Parcellation Toolbox (Fan et al., 2016). We explored treatment-induced changes in rCBF via a mixed 3 × 2 ANOVA with ‘Treatment’ as between-subjects factor (i.e. TAU + MBSR, TAU + FibroQoL and TAU only) and ‘Period’ as within-subjects factor (i.e. baseline and follow-up). We included patients’ ages to the model as an additional nuisance covariate. We examined the Treatment arm × Period interaction effect and explored the results further via post hoc paired *t*-tests (i.e. baseline vs follow-up scans) within each treatment group. We further investigated rCBF changes observed in the amygdala by extracting the mean rCBF value of the amygdala (across the entire ROI) and calculating Pearson’s correlations with self-report measures in SPSS v. 26. We also examined the correlation between mean rCBF in the amygdala at baseline and changes on self-report clinical measures after treatment, as well as correlation between baseline self-report measures and delta rCBF scores in the amygdala (i.e. baseline minus follow-up). For effect size estimation, Hedge’s *g* statistics were calculated as a function of Cohen’s *d* in order to avoid inflation effects due to sample sizes below 20 (Hedges & Olkin, 2014). Finally, we examined the relationship between self-report clinical scores and rCBF across our ROIs by means of a multiple regression analysis in SPM. In this case, a separate model was estimated for each one of the behavioural datasets, using clinical measures scores at baseline and delta scores (i.e. baseline minus follow-up) as regressors. We estimated additional regression models including all participants and a separate intercept per treatment group, in order to look at whether regression slopes (i.e. linear relationship between changes in rCBF and changes in clinical scores) differed across groups.

## Results

### Self-report Clinical Measures

The final sample size was *n* = 14 for the TAU + MBSR group, *n* = 18 for the *TAU* + *FibroQoL* group and *n* = 14 for the TAU only group. At baseline, there were not significant differences across the three treatment groups in terms of age, years from FMS diagnosis, and FIQR, HADS anxiety, PCS and VAS scores. Paired *t*-test results indicated that the TAU + MBSR group experienced a reduction in PCS scores for the *magnification* subscale after treatment (*t*_(13)_ = 2.787, sig = 0.018); however, this result did not survive Bonferroni’s correction for multiple comparisons across all PCS subscales. The *TAU* + *FibroQoL* group presented score reductions in both the *rumination* and *helplessness* subscales of the PCS (*t*_(17)_ = 3.559, sig = 0.003 and *t*_(17)_ = 2.964, sig = 0.009 respectively). There were no significant changes in reported pain, FIQR or HADS measures following treatment across groups. We observed a reduction in reported pain on the control group (*t*_(13)_ = 3.067, sig = 0.009). A summary of all results on self-report measures can be found in Table 1.

**Table 1:** Summary of self-report clinical data and results from paired *t*-test analyses across treatment groups

	**TAU + MBSR** **Baseline (*N* = 14)**	**TAU + MBSR** **Follow-up (*N* = 14)**	**TAU + FibroQoL Baseline (*N* = 18)**	**TAU + FibroQoL** **Follow-up (*N* = 18)**	**TAU** **Baseline (*N* = 14)**	**TAU** **Follow-up (*N* = 14)**
Age	54.15 (± 8.83)	-	52.27 (± 8.68)	-	51.5 (± 8.40)	-
Years from FMS duration	15.54 (± 9.97) m.v. = 2	-	9.76 (± 4.02) m.v. = 1	-	12.45 (± 11) m.v. = 3	-
Mean VAS (SD)	5.74 (± 2.32)	4.99 (± 2.12)	6.04 (± 2.03)	5.61 (± 2.4)	6.84 (± 1.88)	5.15 (± 2.23)
*t* (sig)	0.953 (0.361)		1.023 (0.321)		**3.067 (0.009)**	
Mean FIQR (SD)	61.97 (± 18.02) m.v. = 1	48.79 (± 21.42)	65.75 (± 17.20) m.v. = 1	58.82 (± 21.74)	59.304 (± 27.23)	59.54 (± 24.57)
*t* (sig)	2.115 (0.056)		1.964 (0.067)		− 0.097 (0.924)	
Mean HADS depression (SD)	7.46 (± 4.87)	7.07 (± 4.66)	9 (± 4.66)	7.35 (± 5.43)	8.21 (± 5.10) m.v. = 1	9.38 (± 4.5)
*t* (sig)	0.256 (0.802)		1.679 (0.113)		− 0.705 (0.494)	
Mean HADS anxiety (SD)	9.92 (± 3.72)	9.30 (± 5.64)	12 (± 4.34)	9.94 (± 4.69)	9.14 (± 4.5) m.v. = 1	10.46 (± 3.4)
*t* (sig)	0.387 (0.706)		1.667 (0.115)		− 1.474 (0.166)	
Mean PCS total (SD)	18.66 (± 15.06) m.v. = 1	10.92 (± 8.21)	25.58 (± 14.02)	17.82 (± 13.63)	16.29 (± 10.78)	22.30 (± 14.91)
*t* (sig)	**2.825 (0.015)**		**2.901 (0.010)**		− 1.366 (0.197)	
Mean PCS rumination (SD)	5.16 (± 4.7) m.v. = 1	2.84 (± 2.67)	9.41 (± 4.67)	6.35 (± 4.51)	5.85 (± 4.58) m.v. = 1	7.15 (± 4.77)
*t* (sig)	2.092 (0.06)		**3.559 (0.003)**		− 0.801 (0.439)	
Mean PCS magnification (SD)	4.75 (± 3.59) m.v. = 1	2.69 (± 2.21)	4.94 (± 3.49)	3.88 (± 2.75)	3.64 (± 2.23) m.v. = 1	5.07 (± 3.68)
*t* (sig)	**2.787 (0.018)**		1.394 (0.182)		− 1.921 (0.079)	
Mean PCS helplessness (SD)	8.75 (± 7.67) m.v. = 1	5.38 (± 4.87)	11.23 (± 6.95)	7.58 (± 7.17)	6.78 (± 5.32) m.v. = 1	10.07 (± 7.54)
*t* (sig)	1.924 (0.081)		**2.964 (0.009)**		− 1.334 (0.207)	

*All significant effects but PCS Magnification in MBSR + TAU group were maintained after Bonferroni correction was applied

*TAU*, treatment as usual; *MBSR*, mindfulness-based stress reduction treatment; *FibroQoL*, psychoeducational programme; *VAS*, visual analogue scale; *FIQR*, revised fibromyalgia impact questionnaire; *PCS*, pain catastrophising scale; *m.v.*, missing value. Alpha = 0.05

### Effects of Treatment on rCBF

**Fig. 3:**
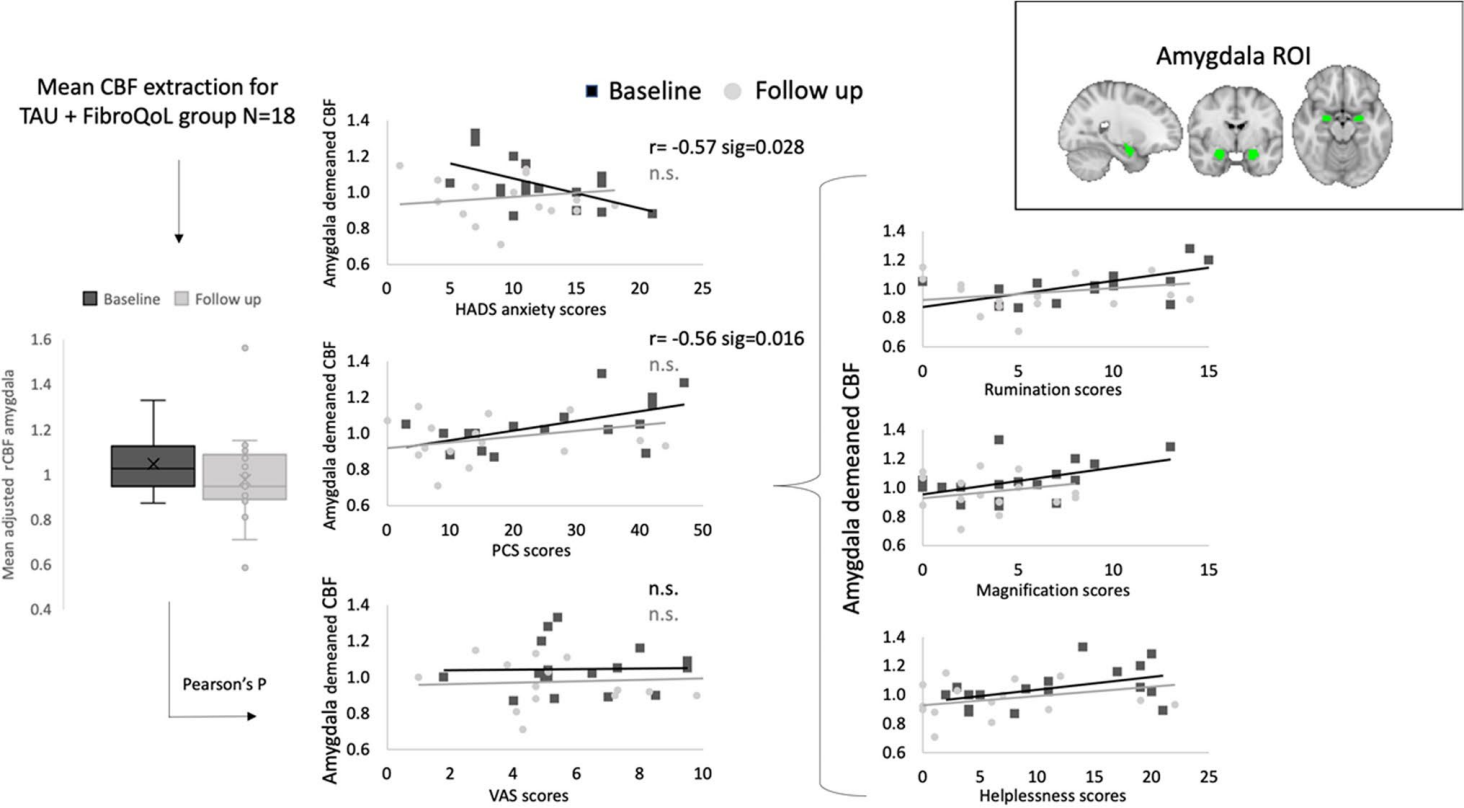
Summary of results from correlation analyses between rCBF in the amygdala bilaterally and behavioural measures for TAU + FibroQoL group. ROI, region of interest. TAU, treatment as usual; FibroQoL, psychoeducational programme; HADS, hospital anxiety and depression scale; PCS, pain catastrophising scale; VAS, visual analogue scale. Alpha = 0.05

We examined changes in rCBF after treatment across all treatment groups. Hypothesis-led analysis revealed a significant interaction effect between ‘Treatment’ and ‘Period’ in the left amygdala (*F* = 9.26, *p*_FWE_ = 0.037, peak coordinates (MNI, x y z) =  − 26 − 4 − 18, 54 voxels). Post hoc pairwise comparisons within each treatment group indicated that this interaction effect was driven by a rCBF decrease after TAU + FibroQoL treatment (*T* = 3.74, *p*_FWE_ = 0.022, peak coordinates (MNI, x y z) =  − 27 − 2 − 21, 58 voxels). Further to these results, we sought to determine whether changes in rCBF in the TAU + FibroQoL group in the amygdala related to a modulation of negative affective comorbid symptoms present in chronic pain, as it has been previously suggested (Neugebauer et al., 2004). Accordingly, we computed Pearson’s correlations between amygdala mean rCBF and both the HADS anxiety subscale and the PCS (Fig. 3). At baseline, mean rCBF in the amygdala correlated negatively with HADS anxiety scores (*r* =  − 0.57, *p* = 0.028) and positively with PCS total scores (*r* = 0.56, *p* = 0.016). Post hoc multiple regression analysis combining the three PCS subscales (i.e. rumination, magnification and helplessness) revealed that baseline rCBF could not be predicted by a linear combination of the subscales (overall model fit *R*^2^ = 0.408, *p* = 0.07) and only the rumination subscale alone at baseline was a significant predictor of amygdala rCBF (overall model fit *R*^2^ = 0.305, *p* = 0.007; CBF_amygdala_ = 0.628 × Rumination + 0.877). None of these correlations reached significance for the follow-up assessment. We found no significant correlation between rCBF in the amygdala and VAS scores in either period. In order to further interpret these results, we investigated whether baseline rCBF in the amygdala for this treatment group could predict changes in PCS and HADS anxiety scores after treatment (i.e. delta scores) and vice versa. Baseline rCBF correlated positively with delta PCS scores (i.e. PCS_baseline_ − PCS_follow up_), that is, greater rCBF levels at baseline corresponded with greater improvement of pain catastrophising (*r* = 0.56, *p* = 0.018). Baseline rCBF did not correlate with delta HADS anxiety measures; however, baseline HADS anxiety scores were positively correlated with delta rCBF (*r* = 0.71, *p* = 0.001), that is, greater anxiety scores corresponded with greater rCBF decreases after TAU + FibroQoL treatment. We observed no significant results for baseline VAS scores or delta VAS scores.

### Regression Analyses

**Fig. 4:**
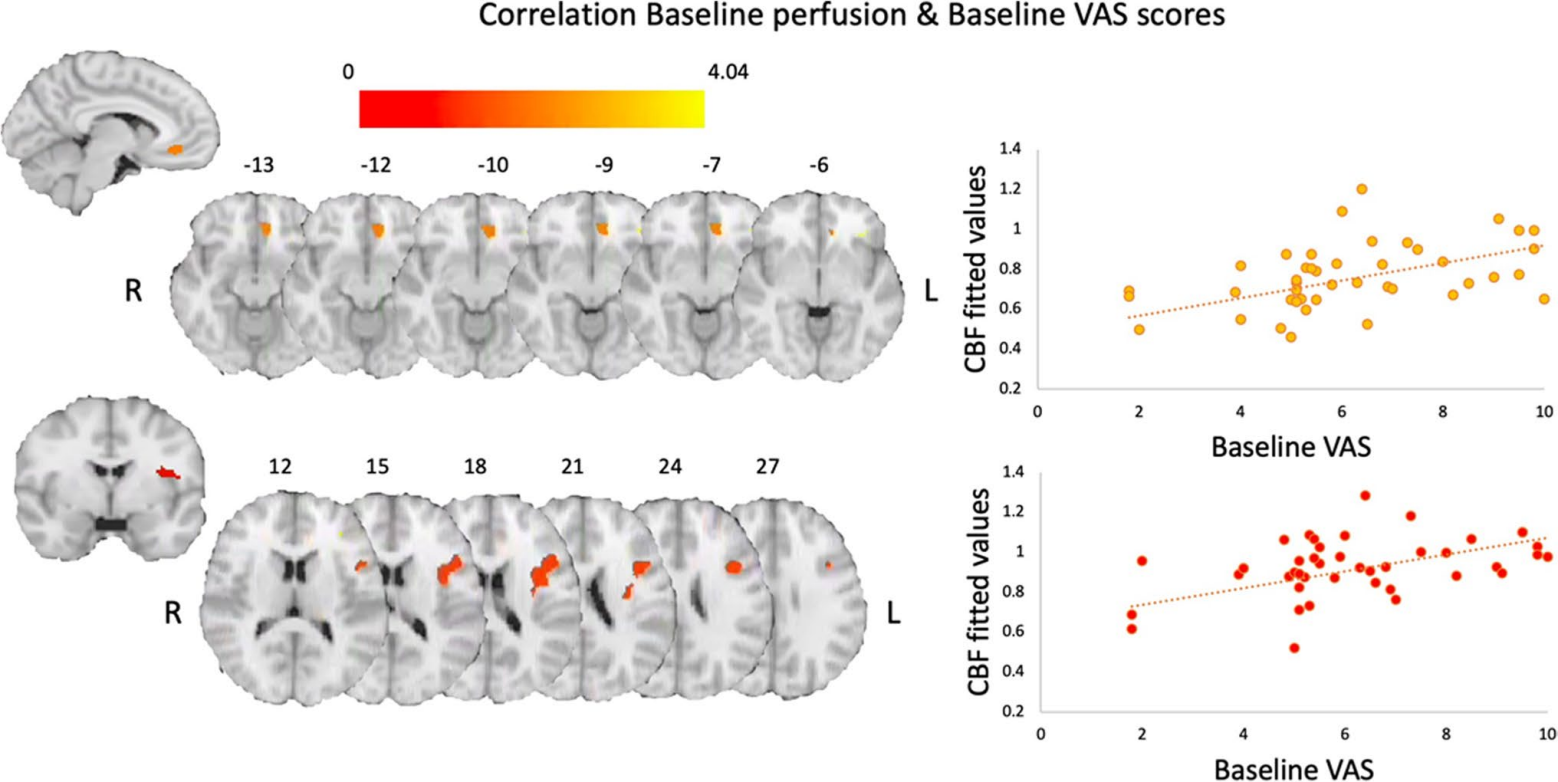
Results from voxel-wise correlation analysis between whole-brain baseline perfusion and baseline VAS scores. Initial height threshold = 0.005. Results are significant at peak level after family-wise error correction at alpha = 0.05 post small volume correction in the subgenual ACC (top panel) and the anterior insula (bottom panel)

**Fig. 5:**
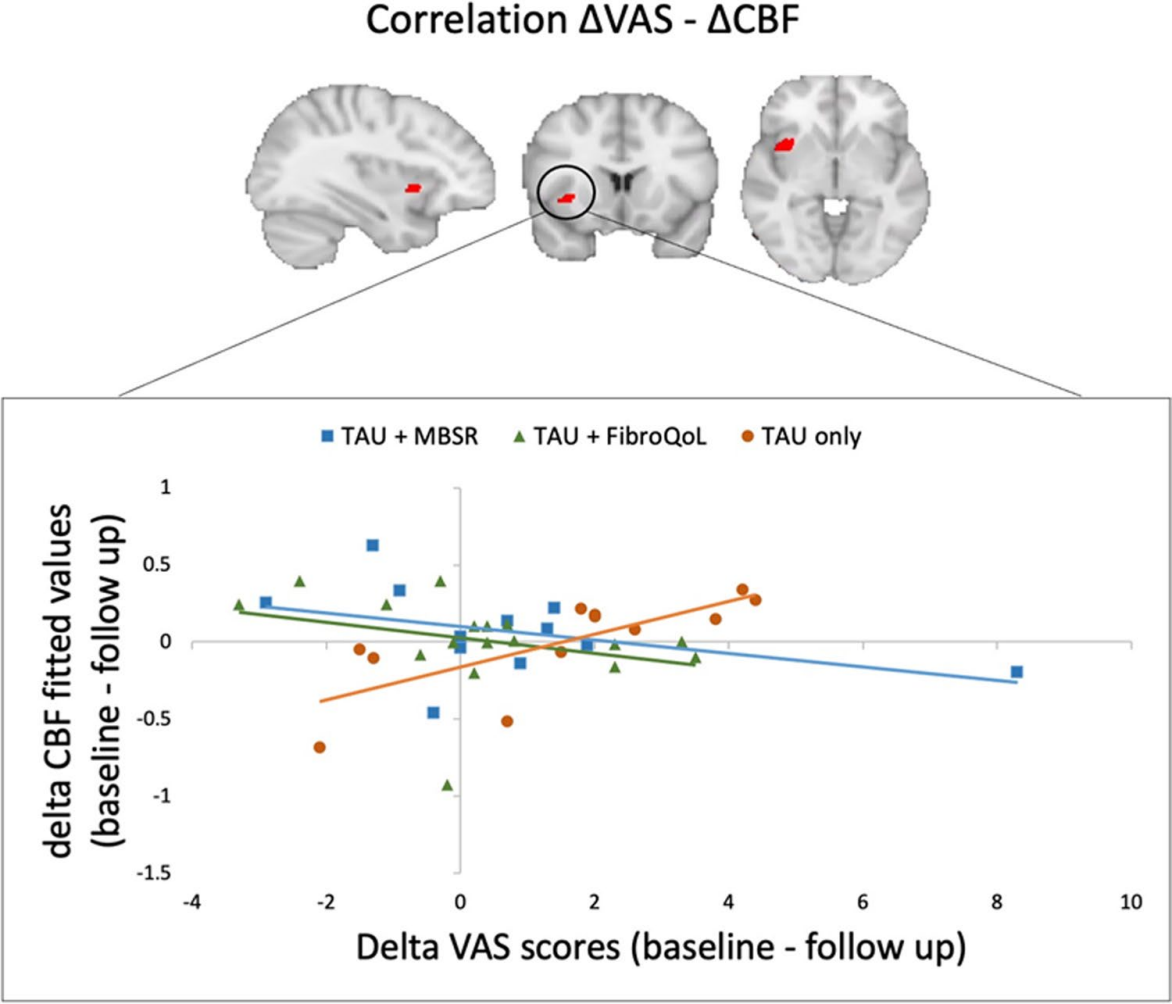
Comparison of regression slopes between delta VAS scores and delta CBF across treatment groups. Initial height threshold = 0.005. Results are significant at peak level after family-wise error correction at alpha = 0.05 post small volume correction in the anterior insula

We investigated how baseline rCBF may relate to any of our self-report measures of interest via multiple linear regression models. We found that baseline VAS scores could predict baseline rCBF levels in the left AI (*T* = 4.20, *p*_FWE_ = 0.026, peak coordinates (MNI, x y z) =  − 36 9 18, 286 voxels) and the left sgACC (*T* = 3.96, *p*_FWE_ = 0.013, peak coordinates (MNI, x y z) =  − 9 34 − 10, 112 voxels) across all participants (Fig. 4). Baseline rCBF did not predict changes in self-report measures after treatment in any of the patient groups; however, the regression slopes for the relationship between delta VAS scores and delta rCBF were significantly different between treatment groups and the control group in the right AI (*T* = 3.68, *p*_(FWE)_ = 0.043, peak coordinates (MNI, x y z) = 44 10 − 6, 85 voxels) where there was a negative relationship between delta VAS and delta rCBF for the TAU + MBSR and TAU + FibroQoL treatments but a positive relationship in the case of the TAU only treatment (Fig. 5). That is, large reductions in perceived pain corresponded to small rCBF reductions for both treatment groups, while large perceived pain reductions were associated with large rCBF reductions for the control group.

## Discussion

This work aimed to further understand the mechanisms of action of two non-pharmacological treatments for FMS delivered to female FMS patients, as indexed by changes in rCBF. We identified that the amygdala is differentially affected by these therapies, experiencing reductions in rCBF after 2 months of FibroQoL training programme but not after MBSR training; we also established a relationship between rCBF in the amygdala and patients’ anxiety and pain catastrophizing within the FibroQoL group. Our second aim was to assess whether rCBF is a good marker of individual differences in FMS symptoms; we found that perceived pain at baseline was positively correlated with the ACC and the AI across patients. However, this relationship was not maintained following treatment. We observed that MBSR and FibroQoL programmes disrupted the positive relationship between changes in pain scores and changes in rCBF following treatment in the insula. Overall, these results provide preliminary evidence of the potential of arterial spin labelled fMRI as a useful tool to study FMS pathophysiology and mechanisms of action of nonpharmacological therapies.

FibroQoL caused reductions in rCBF in the amygdala, unlike MBSR or TAU only. Moreover, changes in amygdala rCBF on the FibroQoL group may relate to a disengagement between negative emotional processing and activity in this area, evidenced by the alteration of the linear relationship between anxiety and catastrophizing scores and rCBF after treatment. In healthy populations, the amygdala is well known for its role in stress-induced analgesia (Tershner & Helmstetter, 2000), pain anticipation and vigilance (Davis & Whalen, 2001), and it is a key node involved in pain processing (Veinante et al., 2013). Perfusion in the amygdala did not correlate with VAS estimates of pain intensity at any point. While this may present as a counterintuitive finding, the complex relationship between the amygdala and clinical pain (Neugebauer, 2007) and depression and anxiety scores in FMS has been previously debated (Pujol et al., 2014). A morphometric study reported GM reductions in the amygdala in FMS patients compared to controls that were not related to pain or disability scores (Burgmer et al., 2009) suggesting that physiological and emotional features in FMS depend upon separate, non-linearly related channels. While we cannot refute the mediating role of the amygdala in pain symptoms via the descending pain limbic pathway to the periaqueductal grey (Linnman et al., 2012), our data suggests that its role in modulating clinical improvement concerns mainly automatic anxiety/stress regulation; speculatively, this regulation may occur via a reorganization of the connectivity with other areas, such as the ventromedial prefrontal cortex due to changes in cognitive control (Kral et al., 2018), the AI as one of the salience network pathways (Menon & Uddin, 2010), and the hippocampus in the context of forming and retrieving memories and learning (Davis & Whalen, 2001). The extent of amygdala modulation also appears to depend on baseline anxiety levels, and greater amygdala rCBF at baseline correlated with higher PCS improvement. Previous evidence suggests that dysfunctional FC between the amygdala and the central executive network is exaggerated by high pain catastrophizing (Jiang et al., 2016). One may therefore speculate that basal amygdala activity marks the modulation capacity of this connectivity pathway due to treatment, ultimately influencing the level of ruminating thoughts on pain.

We did not obtain similar results within the MSBR group. Mindfulness practice can improve the processing of affective stimuli following a short intervention (Allen et al., 2012), evidenced in this study by the reductions in pain catastrophizing levels in the MBSR group; however, reductions in amygdala reactivity to negative stimuli and pain seem to only take place in experienced meditators (Kral et al., 2018; Lutz et al., 2013). It is therefore plausible that changes in the perfusion of the amygdala might start to be visible only at later stages of the intervention. Future imaging studies might shed further light in this direction. We did not find any relationship between FIQR and rCBF. FIQR is considered the gold standard measure of functional impairment in FMS; however, it is worth noting that the FIQR covers a broad spectrum of FMS features (i.e. function, overall impact and symptoms), each one likely specific to different phenotypes of patients as well as separate brain regions and neurophysiological processes (Pérez-Aranda, Andrés-Rodríguez, et al., 2019). Given this, it is a reasonable outcome that rCBF in our three separate ROIs could not capture differences in FIQR after treatment or individual FIQR differences.

Our second objective was to determine whether rCBF could be a sensible predictor of pain in this set of FMS patients. In line with our hypothesis, we observed a positive correlation between VAS pain scores and rCBF in the AI and the sgACC. The AI has been widely known for modulating emotional processing and meaning attribution of pain, and it is involved specifically in integrating emotional, rather than sensory, aspects of pain in FMS (Giesecke et al., 2005). The ACC receives nociceptive input from the amygdala, insula and somatosensory cortices (Bliss et al., 2016) and preclinical studies have shown that it is an important and separate hub for mood disorders associated with chronic pain (Barthas et al., 2015). This indicates that ACC activity may well be linked to changes in psychological symptoms in FMS. In fact, the correlation between pain scores and rCBF in these areas disappeared after treatment, suggesting functional reorganization within the salience and pain networks. In this subsample from the main EUDAIMON trial, there was not a significant reduction in perceived pain after treatment across MBSR and FibroQoL groups. From a therapeutic perspective, this was not the most desirable outcome; however, it is important to note that the purpose of this substudy was not to assess the efficacy of mind–body interventions in chronic pain relief, but rather to investigate these techniques at a mechanistic level. Moreover, the VAS scores reflected perceived pain on the day of the scan, which did not necessarily reflect intervention effects on general pain experience. Notably, our regression slope comparison analysis showed evidence for a shift in the association between changes in pain and changes in rCBF after MBSR and FibroQoL in the AI. In contrast with our initial assumptions, large reductions in pain corresponded to little rCBF reductions after treatment. Although reductions in pain in FMS after a nonpharmacological intervention have been linked to decreased functional connectivity of the AI with the default mode network (Napadow et al., 2012) and the somatosensory cortex (Lazaridou et al., 2017), it is important to note that the role of the AI in acute and chronic pain extends beyond sensory processing; it is a prime node of the salience network (Menon & Uddin, 2010) and is key in the integration of context meaning and emotional states to make decisions on potentially harmful or pain stimuli (Wiech et al., 2010). Moreover, these functions can became increasingly maladaptive with age in chronic pain patients (Ceko et al., 2013). In general, mind–body techniques focus on improving emotion processing and judgment and on eliminating attention biases in relation to our own physiological sensations (Wu et al., 2019), all roles which relate to AI activity. We therefore suggest that rather than ‘reducing hyperactivity’ of the AI, FibroQoL and MBSR treatments provoke a retraining on this area, and while aberrant connectivity with other areas involved in clinical pain may be diminished, greater control over internal and external stimuli discrimination as well improved affective processing leads to a stronger engagement of the AI at rest. This is also evidenced by the fact that although the control group did report reductions in pain symptoms (discussed below), we observed the opposite tendency. In fact, this VAS reduction in the control group was a surprising result. Nevertheless, placebo effects in fibromyalgia RCTs have been reported (Chen et al., 2017), and higher levels of pain catastrophising can enhance this effect (Sullivan et al., 2008). Together with the absence of any significant result at the brain level that relates to treatment efficacy, we argue that this reduction in pain may be the consequence of placebo responses. Another possibility is that this overall reduction in perceived pain ratings is a product of regression to the mean phenomenon, judging by the distribution of baseline VAS scores in the TAU only group (supplementary Fig. 1).

### Limitations and Future Research Directions

We encountered two major difficulties in this study. First, the limited coverage across the brain of the rCBF maps prevented us from adopting a more exploratory, whole-brain analysis approach and forced us to discard a significant proportion of the acquired data. Future research may elucidate whether the distinct brain perfusion patterns across therapies extend to other brain regions. Specifically, we hypothesize that the somatosensory cortex would experience perfusion reductions after mind–body treatment, more so in the case of FibroQoL than following MBSR, since FibroQoL focuses on body relaxation. Similarly, we would expect perfusion in areas focused on top-down pain modulation, such as pain-related nuclei of the brainstem (PAG, VTA, red nucleus, RVM) to experience relative increases following MBSR treatment as a product of improved self-awareness, as well as attentional and cognitive control. The second limitation (and largely a consequence of the first one) is the sample size within each one of our treatment groups. Low sample sizes can affect the reproducibility of results and inflate effect size estimations (Dumas-Mallet et al., 2017). Nevertheless, statistics such as Hedge’s *G* can outperform more ordinary effect size statistics on those cases where group samples are unequal and below 20. Hedge’s *G* values for the present study show that our results yielded medium to large effect sizes (Table 2), providing some confidence on the present results. Future replications of this study on larger samples may shed further light on this matter.

**Table 2:** Summary of effect size calculations

Contrast	*N*	Statistic	Significance	Cohen’s *d*	Hedge’s *G*	Effect
Paired *t*-test (baseline vs follow-up) for TAU + FibroQoL group	18	*T* = 3.74	*p*_FWE_ = 0.022	0.632	0.625	Medium
Correlation between HADS baseline and amygdala baseline CBF	18	*r* = − 0.57	*p* = 0.028	1.387	1.372	Large–very large
Correlation between PCS baseline and amygdala baseline CBF	18	*r* = 0.56	*p* = 0.016	1.351	1.337	Large–very large

*FWE*, family-wise error

## Supplementary Information

Below is the link to the electronic supplementary material.

Supplementary file1 (DOCX 847 KB)

## Declarations

### Data Accessibility Statement

The data that support the findings of this study are available on request from the corresponding author. The data are not publicly available due to privacy or ethical restrictions.

The study was performed in accordance with the ethical standards specified in the 1964 Declaration of Helsinki and its subsequent updates. The FSJD Research Committee Board evaluated and approved the study protocol in May 2015 (PIC-102–15).
