# Supplementary material for: Differential Brain Perfusion Changes Following Two Mind–Body Interventions for Fibromyalgia Patients: an Arterial Spin Labelling fMRI Study
Source: Mindfulness (N Y). 2022 Jan 5;13(2):449–61. doi: 10.1007/s12671-021-01806-2 (PMC8831296; doi:10.1007/s12671-021-01806-2)
Supplement: Supplementary file 1 — Supplementary file1 (DOCX 847 KB) [file 12671_2021_1806_MOESM1_ESM.docx]

**Supplementary information**

**Supplementary Figure 1. Boxplots of behavioural results across treatment groups (baseline vs follow up sessions).** NOTE: Boxplots marked with red squares represent statistically significant paired t-test following multiple comparisons correction (alpha = 0.05).

**
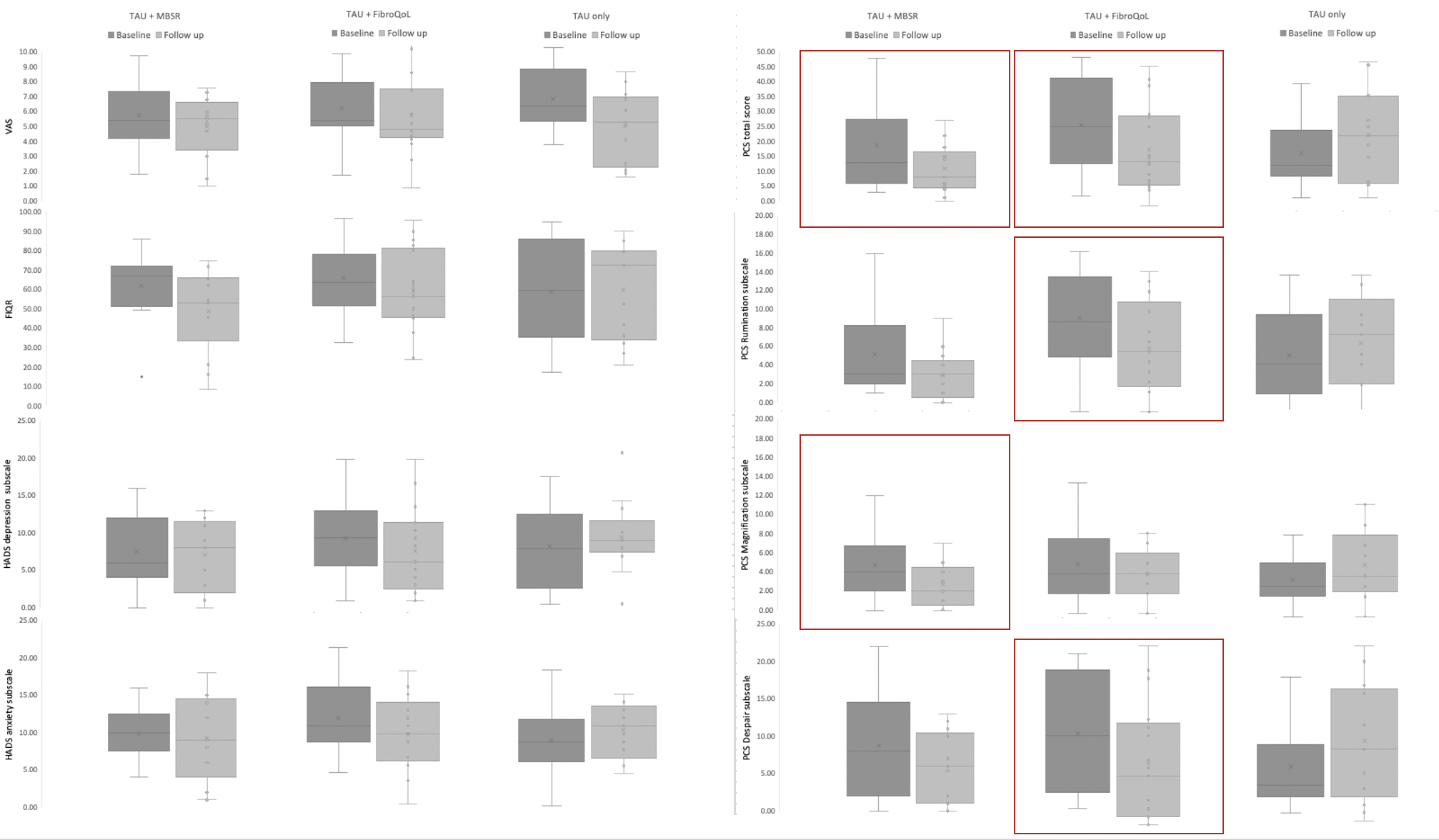
**

**Supplementary Figure 2. Information on treatment as usual across patients in each treatment arm**

|  | | MBSR+TAU | FIbroQoL+TAU | TAU only |
| --- | --- | --- | --- | --- |
| Anti-inflammatory (%) | 30.8 | | 11.1 | 15.4 |
| Opioids (%) | 23.1 | | 38.9 | 30.8 |
| Antiepileptic (%) | 15.4 | | 16.7 | 15.4 |
| Muscle relaxant (%) | 0 | | 5.6 | 0 |
| Antidepressants (%) | 46.2 | | 50.0 | 46.2 |
| Anxiolytics (%) | 15.4 | | 44.4 | 46.2 |
